# Target sites for chemical regulation of strigolactone signaling

**DOI:** 10.3389/fpls.2014.00623

**Published:** 2014-11-05

**Authors:** Hidemitsu Nakamura, Tadao Asami

**Affiliations:** ^1^The Chemical Biology Laboratory, Department of Applied Biological Chemistry, Graduate School of Agricultural and Life Sciences, The University of TokyoTokyo, Japan; ^2^Program of Japan Science and Technology Agency, Core Research for Evolutional Science and TechnologyKawaguchi, Japan; ^3^King Abdulaziz UniversityJedda, Saudi Arabia

**Keywords:** plant growth regulator, strigolactone, biosynthesis inhibitor, agonist, antagonist, 3D structure, *in silico* screening

## Abstract

Demands for plant growth regulators (PGRs; chemicals that control plant growth) are increasing globally, especially in developing countries. Both positive and negative PGRs are widely used to enhance crop production and to suppress unwanted shoot growth, respectively. Strigolactones (SLs) are multifunctional molecules that function as phytohormones, inhibiting shoot branching and also functioning in the rhizospheric communication with symbiotic fungi and parasitic weeds. Therefore, it is anticipated that chemicals that regulate the functions of SLs will be widely used in agricultural applications. Although the SL biosynthetic pathway is not fully understood, it has been demonstrated that β-carotene isomerases, carotenoid cleavage dioxygenases (CCDs), and a cytochrome P450 monooxygenase are involved in strigolactone biosynthesis. A CCD inhibitor, abamine, which is also an inhibitor of abscisic acid biosynthesis, reduces the levels of SL in several plant species and reduces the germination rate of *Orobanche minor* seeds grown with tobacco. On the basis of the structure of abamine, several chemicals have been designed to specifically inhibit CCDs during SL synthesis. Cytochrome P450 monooxygenase is another target enzyme in the development of SL biosynthesis inhibitors, and the triazole-derived TIS series of chemicals is known to include SL biosynthesis inhibitors, although their target enzyme has not been identified. Recently, DWARF14 (D14) has been shown to be a receptor for SLs, and the D-ring moiety of SL is essential for its recognition by D14. A variety of SL agonists are currently under development and most agonists commonly contain the D-ring or a D-ring-like moiety. Several research groups have also resolved the crystal structure of D14 in the last two years. It is expected that this information on the D14 structure will be invaluable not only for developing SL agonists with novel structures but also in the design of inhibitors of SL receptors.

## INTRODUCTION

Chemicals are widely used in agriculture to increase the yields of crops. For example, pesticides, including herbicides, fungicides, insecticides, and/or insect growth regulators, protect crops from the attack of pests that damage them, such as weeds, fungal diseases, and insects. Because pesticides usually protect crops by killing these pests, they are thought of as negative regulators of pests. However, because plant growth regulators (PGRs) are chemicals that control plant growth and benefit crop production by enhancing crop quantities and quality and by improving the postproduction quality of some plants, they are thought of as positive regulators of plants. In developing countries, such as China, the plant growth regulator industry has seen remarkable progress and shows attractive future market pot ential (http://www.reuters.com/article/2010/05/28/idUS145314+28-May-2010+BW20100528). It is likely that PGRs will be utilized for large numbers of species and cultivars.

The most popular target of PGRs is gibberellin (GA) biosynthesis. In this case, PGRs are considered plant growth retardants and are applied to agronomic and horticultural crops to reduce unwanted longitudinal shoot growth without lowering plant productivity (Rademacher, 2000). Their targets are copalyl-diphosphate synthase and *ent*-kaurene synthase, which are involved in the early steps of GA metabolism; cytochrome P450-dependent monooxygenases, which are involved in the oxidization of *ent*-kaurene to *ent*-kaurenoic acid; and dioxygenases, which catalyze the late steps of GA formation by mimicking 2-oxoglutarate (Rademacher, 2000). Enzymes similar to those involved in GA biosynthesis also play important roles in the formation of strigolactones (SLs), brassinosteroids, abscisic acid, and other plant hormones.

The importance of the chemicals that control plant function has recently been increasing, not only in agriculture but also in the plant sciences. The primary advantage of using bioactive chemicals to analyze the roles of plant hormones in plants, rather than plant-hormone-deficient mutants, is that they can be applied regardless of the plant species. The phenotypic changes induced by chemical treatments can reveal the physiological functions associated with the target proteins. Furthermore, genetic redundancy does not significantly influence the effects of these inhibitors. Plants treated with an antagonist or an inhibitor of biosynthesis show phenotypes almost identical to those of untreated plants, as is seen in multiple mutants when the target protein is redundant. Finally, chemicals easily regulate the functions of their target proteins only transiently (Kitahata and Asami, 2011). Therefore, the utilization of biosynthesis inhibitors or receptor inhibitors is a useful alternative to mutations for dissecting biological processes (Blackwell and Zhao, 2003).

Recently, the scientific discipline of chemical biology has increased in popularity. The goal of chemical biology is to clarify biological mechanisms using small bioactive organic compounds. In plant hormone research, the increasing use of chemical-biology-based methods has been very effective. For example, with plant hormone biosynthesis inhibitors, researchers can create plant hormone deficiencies in specific plants and under specific conditions. For instance, molecular genetic studies have been conducted using inhibitors of plant hormone biosynthesis to select mutants that are resistant to those inhibitors. This approach has been very successful, especially in brassinosteroid research, in which the brassinosteroid biosynthesis inhibitor Brz was used to isolate the Brz-resistant mutant *bzr1* to identify the novel protein BZR1, which functions in the brassinosteroid signal transduction pathway (Wang et al., 2002).

**FIGURE 1:**
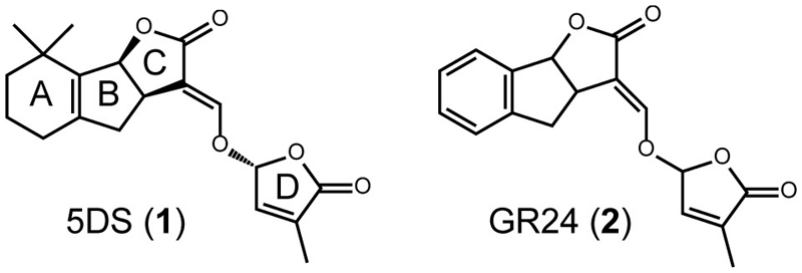
**Structures of SLs.** Structure of a natural SL, 2′-*epi*-5-deoxystrigol (5DS; 1), and a synthetic SL, GR24 (2).

Strigolactones are terpenoids that contain a lactone ring in their molecules, and are produced in a variety of plant species (compound 1 in **Figure 1**). They are multifunctional molecules, acting as germination stimulants in root parasitic weeds, root-derived signals that induce hyphal branching in arbuscular mycorrhizal fungi, and plant hormones that regulate various phenomena, such as shoot branching, root morphology, secondary growth, and so on (Cook et al., 1966; Akiyama et al., 2005; Gomez-Roldan et al., 2008; Umehara et al., 2008; Seto et al., 2012). Several branching mutants have been identified as mutants of SL biosynthesis and signaling. At present, two carotenoid cleavage dioxygenases (CCDs; AtMAX3 and AtMAX4), one carotenoid isomerase (AtDWARF27 (AtD27), and one cytochrome P450 (AtMAX1) are known to be involved in the biosynthesis of SLs in *Arabidopsis*. AtMAX2 and AtDWARF14 (AtD14), an F-box protein and an α/β hydrolase, respectively, act in SL signaling (Waters et al., 2012a,b). A screen for genetic suppressors of *Atmax2* mutant identified that members of SMXL protein family act downstream of AtMAX2 in SL signaling (Stanga et al., 2013). More recently, DWARF53 (D53), a member of the SMXL protein family in rice, was reported to be a substrate of the SCF^D3^ complex and rapidly degraded in the presence of SL. These data suggest that D53 is a repressor of SL signaling (Jiang et al., 2013; Zhou et al., 2013).

As described above, chemicals that regulate the functions of SLs will be very useful, so several approaches have been used to develop chemically synthesized SL agonists. As a consequence, several chemicals have been reported that are germination stimulants of root parasitic weeds, regulators of shoot branching, and inducers of hyphal branching in arbuscular mycorrhizal fungi (Akiyama et al., 2010; Yoneyama et al., 2010; Zwanenburg and Posplsil, 2013). One of the great successes in this field has been the identification of GR24 (coumpound 2 in **Figure 1**) by Zwanenburg and Posplsil (2013). GR24 (2) is now widely used as a standard mimic in SL research and is known to be more stable than natural SLs (Akiyama et al., 2010). However, there has been no report of any antagonists of SLs. Because D14 has been identified as an SL receptor (Jiang et al., 2013; Nakamura et al., 2013; Zhou et al., 2013) and its three-dimensional (3D) structure is available (Hamiaux et al., 2012; Kagiyama et al., 2013; Nakamura et al., 2013; Zhao et al., 2013), the design and development of SL agonists and antagonists will be a fascinating target for chemists.

**FIGURE 2:**
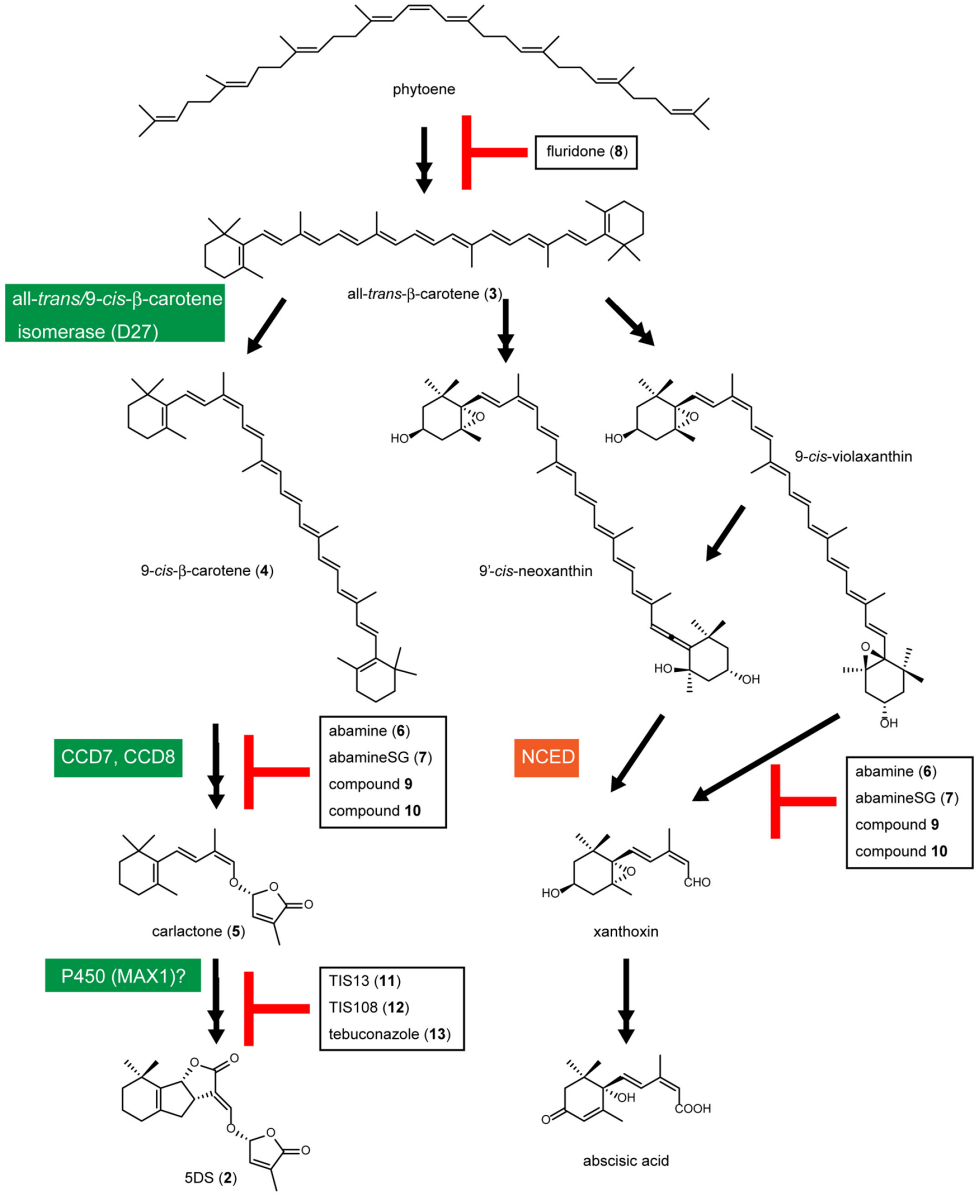
**Predicted biosynthetic pathway of SLs and abscisic acid.** In the SL biosynthetic pathway, D27 catalyzes the isomerization of all-*trans*-β-carotene (3) to 9-*cis*-β-carotene (4), and 9-*cis*-β-carotene (4) is converted to carlactone (5) by CCD7 and CCD8. Carlactone (5) would be oxidized by some enzymes including a cytochrome P450 monooxygenase MAX1 (CYP711A) and converted to SLs including 5DS. Abscisic acid is also synthesized from all-*trans*-β-carotene (3). In the abscisic acid biosynthetic pathway, 9-*cis*-epoxycarotenoid dioxygenase (NCED) catalyzes the cleavage of 9-*cis*-epoxycarotenoid at the 11–12 double bond to produce a precursor of ABA, xanthoxin.

**FIGURE 3:**
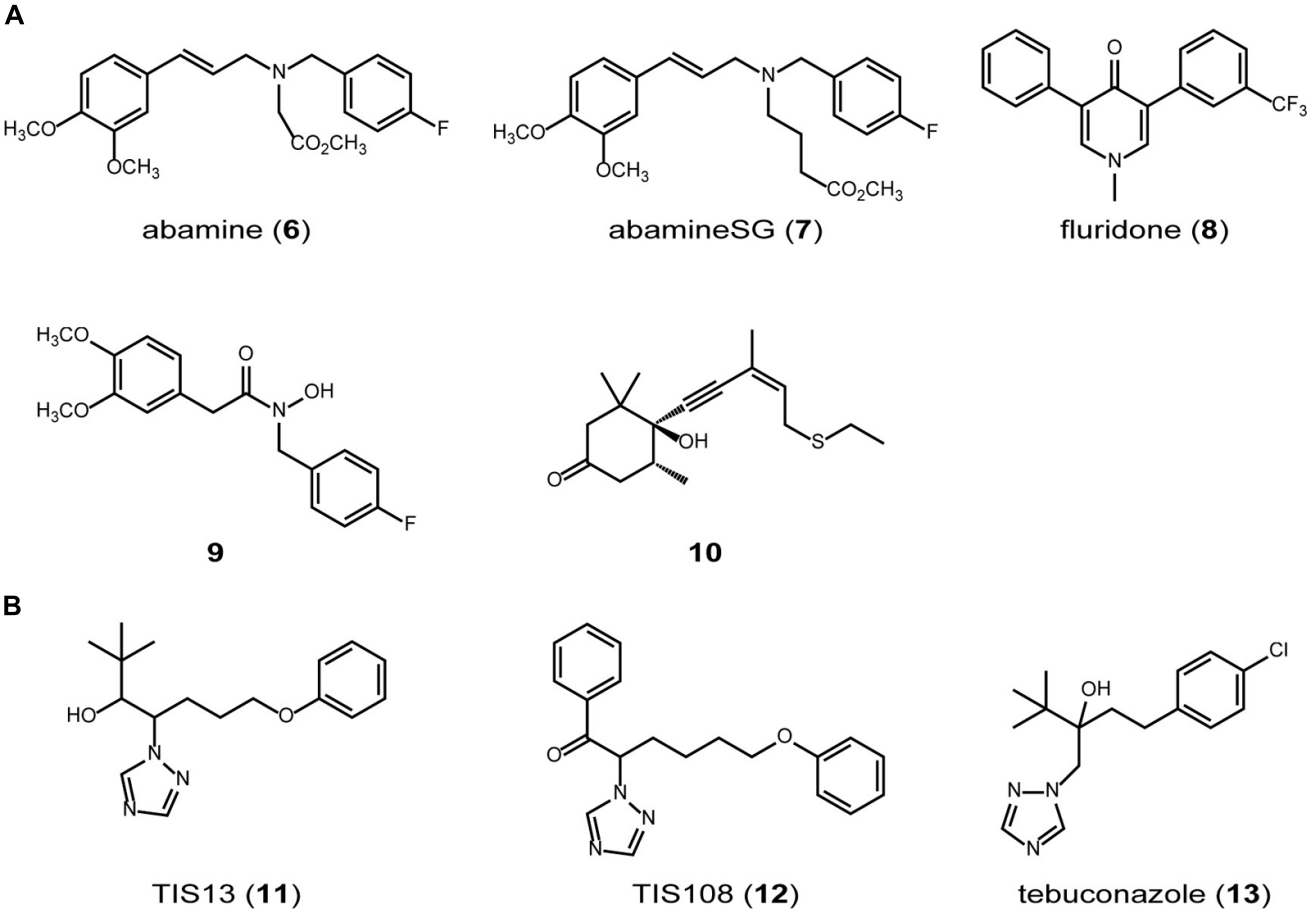
**SL biosynthesis inhibitors.** (A) carotenoid synthesis/cleavage inhibitors. (B) cytochrome P450 inhibitors.

The SL biosynthetic pathway is not yet fully understood. However, the involvement of Fe-containing β-carotene isomerase (D27 in rice), CCD7, CCD8, and cytochrome P450 monooxygenase (MAX1 in *Arabidopsis*) in SL biosynthesis has been demonstrated (Seto et al., 2012). Alder et al. (2012) demonstrated that conversion of all-*trans*-β-carotene (3) to 9-*cis*-β-carotene (4) by D27 and cleavage of 9-*cis*-β-carotene (4) by CCD7 and CCD8 generates carlactone (5), which has a carbon skeleton similar to that of the SLs, including a methylbutenolide ring, a characteristic part of the SL structure (**Figure 2**). Because all of these enzymes include an iron atom in their molecules and nitrogen has a lone pair electrons that can form a coordinated bond with the 3D orbital of the iron atom, chemicals that include a nitrogen atom(s) in their molecules and have binding affinity for the substrate-binding pocket of these enzymes could be inhibitors of SL biosynthesis. For example, abamine (6), the first CCD inhibitor, which includes a nitrogen in its molecule, inhibits 9-*cis*-epoxycarotenoid dioxygenase (NCED) in the abscisic acid biosynthetic pathway and reduces abscisic acid levels in abamine (6)-treated *Arabidopsis* (**Figures 2 and 3A**; Han et al., 2004a,b). 1H-1,2,4-triazole or 1H-1,3-imidazole derivatives, such as uniconazole-P and paclobutrazol, inhibit a variety of members of the cytochrome P450 enzyme group. The triazole or imidazole moiety is a key component in the action of cytochrome P450 inhibitors because the nitrogen atoms in these groups are essential for binding the heme iron in the cytochrome P450s. In this paper, we review the recent research into the regulators of SL functions, including SL biosynthesis inhibitors and agonists, and the possibility of finding SL antagonists based on the 3D structure of the SL receptor D14.

## SL BIOSYNTHESIS INHIBITORS

Lignostilbene-alpha,beta-dioxygenase cleaves the olefinic double bond of phenolic stilbenes with a mechanism similar to that of NCED, a key enzyme in abscisic acid biosynthesis (**Figure 2**). Han et al. synthesized several analogs of stilbene and found that several types of lignostilbene analogs that contain nitrogen in the C–C bond are inhibitors of lignostilbene-alpha,beta-dioxygenase (Han et al., 2002, 2003). On the basis of these findings, we started to design and synthesize NCED inhibitors and identified abamine (6) as a specific inhibitor of abscisic acid biosynthesis, targeting NCED (**Figures 2 and 3A**; Han et al., 2004a,b). A structure-activity relationship study of abamine (6) identified a more potent and specific NCED inhibitor, abamineSG (7; **Figures 2 and 3A**). Abamine (6) and abamineSG (7) contributed significantly to the recent findings that abscisic acid plays a role in controlling the number of nodules on the roots of leguminous plants (Han et al., 2004a; Suzuki et al., 2004) and in the high-light response of *Arabidopsis* (Galvez-Valdivieso et al., 2009). Fluridone (8) inhibits the biosynthesis of all carotenoids and carotenoid-derived metabolites (**Figures 2 and 3A**). Because carotenoid biosynthesis is necessary for the biosynthesis of normal levels of SLs (Matusova et al., 2005; López-Ráez et al., 2008), fluridone (8) may also be an inhibitor of SL biosynthesis. However, because fluridone (8) causes the photodestruction of chlorophyll and lethal damage to cells, it is not an ideal inhibitor with which to study the biological roles of SLs in plants. We consider that specific SL biosynthesis inhibitors could be useful tools for the biochemical analysis of SL biosynthesis and could extend our understanding of the biological roles of SLs in plants. In this context, we have begun to search for inhibitors of SL biosynthesis (**Figure 3A**).

9-*cis*-epoxycarotenoid dioxygenases belong to the family of CCDs and control the rate-limiting step in the abscisic acid biosynthetic pathway in plants (Iuchi et al., 2001). It has been suggested that abamine (6) also targets other CCDs (Kitahata et al., 2006), but the potency of abamine (6) in the regulation of SL production is yet to be determined. Because CCD7 and CCD8 are involved in the SL biosynthetic pathway and share similar functions and sequences with all the CCDs, abamine (6) might affect SL biosynthesis by inhibiting CCD7, CCD8, or other related enzymes in addition to NCED. It therefore has potential as a new scaffold for the development of regulators of SL biosynthesis. In this context, we evaluated the potency of abamine (6) in the regulation of SL production. We found that abamine (6) reduces the levels of SLs in several plant species and the germination rate of *Orobanche minor* seeds grown with tobacco (Kitahata et al., 2011). Taken together, these data suggest that abamine (6) can be used as a scaffold for the development of specific regulators of SL production. The actual structure of abamine (6) offers clues to the design of new CCD inhibitors. Hydroxamic acid analogs (9; **Figure 3A**), which were designed based on the structures of abamine (6) and abamineSG (7), inhibited the activities of many CCDs, including AtCCD7, and increased the number of branches in inhibitor-treated *Arabidopsis* at a concentration of 100 μM (Sergeant et al., 2009). Similarly, sesquiterpene-like inhibitors of NCED (10; **Figure 3A**) have been designed based on the structure of abamine (6; Boyd et al., 2009). Therefore, abamine (6) can be used as a scaffold for designing new inhibitors targeting several types of CCDs, including CCD7 and CCD8, and CCDs may be good targets for designing and developing specific inhibitors of SL biosynthesis.

Another target enzyme class that may be useful in developing SL biosynthesis inhibitors is the cytochrome P450 monooxygenases. At least one cytochrome P450 monooxygenase (CYP711A) is probably involved in the SL biosynthetic pathway in *Arabidopsis* (Booker et al., 2005) and there are five CYP711 family members in rice (Nelson et al., 2004). We screened a chemical library of triazole derivatives, constructed in our laboratory by Min et al. (1999) and Sekimata et al. (2001, 2002), to find new SL biosynthesis inhibitors that induce tiller bud outgrowth in rice seedlings (**Figure 3B**). We selected TIS13 (11) as a candidate inhibitor of SL biosynthesis (Ito et al., 2010). TIS13 (11) reduced the levels of SL in both the roots and root exudates, and TIS13-induced second tiller bud outgrowth was suppressed by its coapplication with 1 μM GR24 (2). Furthermore, the root exudates of rice treated with 10 μM TIS13 (11) had less germination-stimulating activity on the root parasitic weed *Striga hermonthica* than those of the control plants. These results strongly suggest that TIS13 (11) inhibits SL biosynthesis in rice. Because we considered TIS13 (11) a useful lead compound for developing specific and potent SL biosynthesis inhibitors, we performed a structure–activity relationship study of TIS13 (11) using chemical modification, which led to the identification of the more potent SL biosynthesis inhibitor, TIS108 (12; Ito et al., 2011, 2013a). Besides the TIS series (11 and 12), we found that the fungicide tebuconazole (13), which targets cytochrome P450 in fungi, is also a potent SL biosynthesis inhibitor (Ito et al., 2013b). These results strongly suggest that chemicals targeting cytochrome P450 are good scaffolds upon which to design and develop inhibitors of SL biosynthesis.

Because the TIS series (11 and 12) and tebuconazole (13) are triazole-type inhibitors and have potential affinity for the cytochrome P450s, their target could be CYP711A (**Figure 2**). However, several biosynthetic steps in the SL biosynthetic pathway have still to be clarified and other P450s may be involved in SL biosynthesis. The target site of the TIS series will be identified in future studies.

## DESIGN OF SL AGONISTS AND ANTAGONISTS

Precise knowledge of the mechanism of molecular SL recognition will greatly assist chemists in designing and developing SL agonists and antagonists. Numerous studies have revealed that SL is received by D14 class of α/β hydrolase proteins in rice (Arite et al., 2009; Liu et al., 2009; Kagiyama et al., 2013; Nakamura et al., 2013), *Arabidopsis* (Waters et al., 2012b; Zhao et al., 2013; Chevalier et al., 2014) and petunia (Hamiaux et al., 2012). Karrikins are smoke-derived compounds that stimulate seed germination, and karrikin signals, which probably mimic unknown endogenous signals, are closely related and partially overlapped with SL signals (Waters et al., 2014). Karrikin is recognized by KAI2, a close relative of D14, in *Arabidopsis* (Guo et al., 2013). While numerous lines of evidence indicate that D14 and KAI2 are receptor proteins of SLs and karrikins, respectively, Toh et al. (2014) recently showed that GR24 binds KAI2 as well as D14, suggesting that KAI2 may perceive SLs. At present, several groups have resolved the crystal structure of D14 (Hamiaux et al., 2012; Kagiyama et al., 2013; Nakamura et al., 2013; Zhao et al., 2013), and have made it available on the Protein Data Bank (PDB: 4DNP and 4DNQ; 4IH1, 4IH4, 4IH9, and 4IHA; 3W04, 3W05, and 3W06; 3VXK and 3WIO). However, D14 has a unique mechanism of SL recognition, in that its enzymatic activity hydrolyzes its ligand molecule, SL. In a recent paper, we reported that rice D14 hydrolyzes SLs to produce D-OH, and that D14 then forms a complex with D-OH that is important for its interaction with a rice DELLA protein, SLR1 (Nakamura et al., 2013). Since DELLA proteins are key regulators of GA signaling, this SL-dependent D14–SLR1 interaction is presumed to mediate the crosstalk between SL and GA signaling, although the genetic data to support this interaction is absent at present. In the D14–D-OH crystal, D-OH is located at a site far from the catalytic triad, Ser147–His297–Asp268, and is surrounded by Val194, Ser270, and several aromatic residues, including Phe186, Trp205, Tyr209, and Phe245. These aromatic residues allow favorable hydrophobic and/or van der Waals interactions with D-OH. Mutations at Phe186, Trp205, and Phe245 extinguish the D14–SLR1 interaction, supporting the idea that the D14–D-OH interaction is required for the D14–SLR1 interaction. Therefore, we assume that D-OH is a strong candidate for an active form of SL and designate it “branin” (branching inhibitor).

**FIGURE 4:**
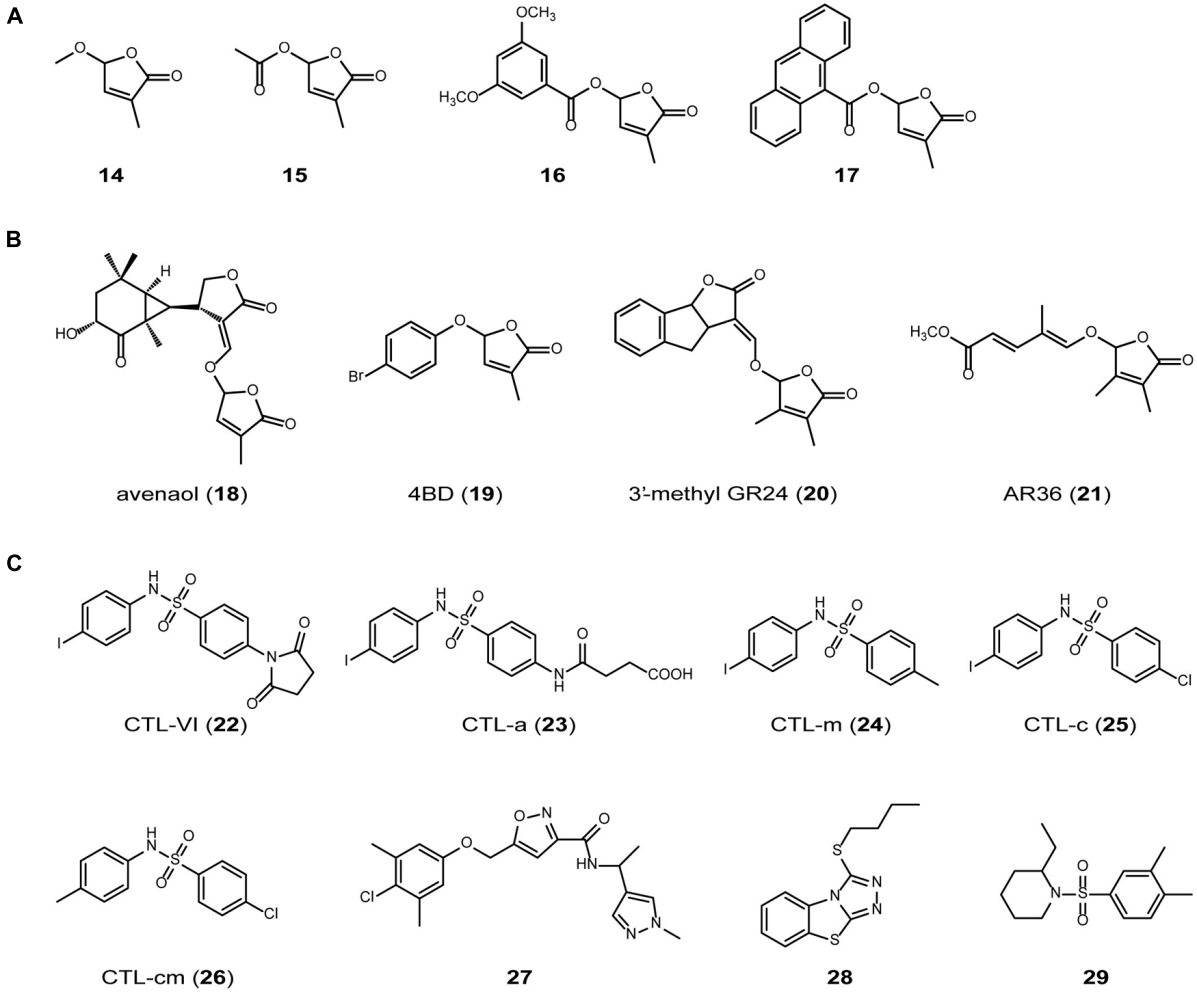
**SL agonists.** (A) SL homologs that are expected to be hydrolyzed by D14 and have tillering inhibition activity in rice. (B) SL mimics reported recently (Fukui et al., 2011, 2013; Boyer et al., 2014; Kim et al., 2014). (C) SL agonists without butenolide miety (Toh et al., 2014).

On the basis of the results discussed above, it is assumed that the structural requirements for active SL are as follows: (1) it must be hydrolyzable by D14; and (2) it must produce D-OH after hydrolysis. These requirements are consistent with the hypothesis proposed by Boyer et al. (2012) on the basis of laborious structure–activity relationship analyses, that the presence of a D-ring is essential for the hormonal activity of SL. To confirm this hypothesis, we prepared various SL homologs (14–17) that are expected to be hydrolyzed by D14 to induce the D14–SLR1 interaction, and determined their ability to inhibit rice tillering (**Figure 4A**; Nakamura et al., 2013). A strong relationship was observed between their tillering inhibition activity and the induction of the D14–SLR1 interaction, suggesting that the yeast two-hybrid system is a useful tool for screening SL agonists. We could not perform enzymatic assays of all the SL homologs because we encountered several difficulties, e.g., stability of the reaction buffer, detectability of the reaction products, etc. Therefore, further studies are required to test this hypothesis more rigorously.

As described above, the sequential conversion of all-*trans*-β-carotene (3) by D27, CCD7 and CCD8 generates carlactone (5), which has a carbon skeleton similar to that of the SLs, including a methylbutenolide ring, a characteristic part of the SL structure (**Figure 2**). When applied exogenously, carlactone (5) rescued the shoot-branching phenotype of *d27* and *d10*, but not that of *d3*, suggesting that carlactone is a biosynthetic intermediate of the SLs (Alder et al., 2012). Supporting this hypothesis, Seto et al. (2014) demonstrated that carlactone (5) is transformed into 2′-*epi*-5-deoxystrigol (1) when ^13^C-labeled carlactone is fed to rice (**Figure 2**). However, it is still possible that carlactone (5) itself is recognized and hydrolyzed by D14 to produce D-OH and elicit SL activity, because carlactone (5) has a methylbutenolide ring connected to a carbon chain *via* an enolether moiety, which can be hydrolyzed to yield D-OH, as shown above. Recently, avenaol (18) was reported to mimic SLs in stimulating the germination of root parasitic plants (**Figure 4B**; Kim et al., 2014). Avenaol (18) lacks the B-ring and has an additional carbon atom between the A- and C-rings. However, it contains the C–D moiety of the SLs, the structural feature common to all known SLs. The plant hormonal activity of avenaol (18) has not yet been determined, but we assume that it is active because its molecule contains a C–D moiety that is hydrolyzed by D14.

Debranones are phenoxy furanone derivatives that inhibit the outgrowth of tillering buds in rice (Fukui et al., 2011). Like SLs, debranones have a D-ring [3-methylfuranone-2(5H)-one] but they lack an enolether moiety. A structure–activity relationship study showed that 5-[4-bromophenoxy]-3-methylfuranone-2(5H)-one (4BD, 19) had similar activity to that of GR24 (2) in many aspects of a biological assay in plants, but is far less active in inducing seed germination in parasitic weeds (Fukui et al., 2013). This suggests that the structural requirements for its hormonal activity in plants and for its activity in the rhizosphere differ, and that 4BD (19) could be useful for controlling the plant architecture without inducing the growth of parasitic weeds.

Recently, Boyer et al. (2014) reported another type of SL analog, including 3′-methyl-GR24 (20) and AR36 (21; **Figure 4B**), which have a dimethylbutenolide motif instead of the D-ring of SL. 3′-methyl-GR24 (20) and AR36 (21) show hormonal activity in pea, but not in parasitic weed germination or fungal hyphal growth, indicating that the dimethylbutenolide structure is recognized by the SL receptor involved in its branching-inhibition activity, but not by the receptor involved in the action of SL in the rhizosphere (Boyer et al., 2014). This suggests that, as well as 4BD (19), dimethylbutenolide-containing compounds are useful for controlling the plant architecture without inducing the growth of parasitic weeds.

Cotylimide (CTL) compound (CTL-VI; 22; **Figure 4C**) was firstly identified as a molecule that increases SL synthesis and regulates light adapted growth through AtMAX2 in *Arabidopsis* (Tsuchiya et al., 2010). Recently, Toh et al. (2014) demonstrated that CTL-VI (22) and its analogs (23–26; **Figure 4C**) bind KAI2 and promote interaction between KAI2 and AtMAX2. These CTL compounds inhibits hypocotyl growth of *Arabidopsis*. These results indicate that they are SL agonist. Toh et al. (2014) also screened 4,182 small molecules to identify novel compounds that promote KAI2-AtMAX2 interaction using the yeast two-hybrid system and obtained three lead compounds (27–29; **Figure 4C**). These three compounds showed SL activity in *Arabidopsis* hypocotyls and *Striga* seed germination assay. Although the germination stimulation activity of these lead compounds was much weaker than that of GR24, this approach can be useful to identify novel SL agonists.

With the recent increase in computer power and the development of bioinformatics algorithms, computer-assisted drug design, which utilizes the 3D structures of proteins determined with X-ray crystallography, has become a common method of drug discovery. Because we now have considerable information on the 3D structure of D14, we are planning to use an *in silico* drug design method to screen for novel agonists and antagonists of D14. For this purpose, we are trying the *in silico* screening of ligands of D14 by using the 3D structure of D14 complexed with D-OH (PDB: 3WIO). Using the LigandScout software (Wolber and Langer, 2005; Wolber et al., 2007), we have selected candidate chemicals on the basis of a ligand-based pharmacophore model, with reference to the structural information for the D14–D-OH complex (PDB: 3WIO). Their structures are quite different from those of known SLs, indicating that SL agonists and/or antagonists with novel structures can be obtained with this *in silico* screening method.

## CONCLUDING REMARKS

In this review, we have described recent attempts to design inhibitors of SL biosynthesis based on the recently accumulating knowledge of the enzymes involved in SL biosynthesis, and to design SL agonists and SL antagonists based on our recent model of SL recognition in plants. These chemicals have potential utility in both agricultural applications and in basic science, to dissect the mechanisms underlying the wide spectrum of SL functions. The stability of these chemicals is an important feature of their agricultural use. Boyer et al. (2014) has developed stable SL mimics by substituting the D-ring with a dimethylbutenolide moiety, but its structure is not very different from that of the D-ring. For almost all the chemicals that mimic SL activities, a D-ring or its derivative is necessary for their SL-like activities. The single possible exception may be CTL (Tsuchiya et al., 2010; Toh et al., 2014), which has no butenolide moiety in its molecule but shows SL-like activity. Although its potency in binding D14 and inducing the D14–D53 and D14–DELLA interactions is not yet clear, the investigation of these characteristics will be interesting.

In addition to the trials described above, we anticipate that the protein–protein interactions necessary for SL signaling may be alternative and efficient targets for the design of novel inhibitors of SL functions. There are many examples of the pharmacological screening of inhibitors of protein–protein interactions (Wilson, 2009), although inhibitors of such interactions are not common among PGRs.

It has been reported that petunia DAD2, a petunia homolog of D14, interacts with petunia MAX2 (Hamiaux et al., 2012) and that *Arabidopsis* MAX2 interacts with BES1 and BZR1 (Wang et al., 2013), which are major brassinosteroid signaling factors. Therefore, the application of inhibitors of SL receptors might have pleiotropic effects on plant growth. In this context, protein–protein interactions, such as the SL-dependent D14–D53 interaction, will be good targets for the regulation of plant growth by chemicals.

## Conflict of Interest Statement

The authors declare that the research was conducted in the absence of any commercial or financial relationships that could be construed as a potential conflict of interest.
